# Monitoring and Analysis of Venture Capital and Corporate Fraud Based on Deep Learning

**DOI:** 10.1155/2022/4589593

**Published:** 2022-05-31

**Authors:** Ruijun Zhang, Lina Zheng

**Affiliations:** School of Business, Renmin University of China, Beijing 100872, China

## Abstract

With the continuous expansion of global investment institutions, the development of the investment industry is gradually accelerating, but the risks behind the investment are also constantly increasing. Using the data of A-share companies in China's capital market from 2010 to 2019, this paper studies the impact of venture capital on corporate fraud. Empirical results show that venture capital holdings reduce the probability and frequency of corporate fraud. These findings remain robust after mitigating endogeneity using PSM, Heckman's two-step, one-step approach, suggesting a causal relationship between venture capital holdings and fraud reduction. Further research shows that the way venture capital holdings reduce corporate fraud is to suppress corporate fraud by improving the company's internal and external information environment. Furthermore, venture capital holdings play an important role in the governance of corporate disclosure fraud and operational fraud, but not in the governance of TMT fraud. In addition, the venture capital has better inhibitory effects on the supervision and governance of the fraud frequency of nonstate-owned enterprises compared with state-owned enterprises. The results of this research imply that venture capital shareholding plays an important role in preventing corporate fraud. This study contributes to the researches about the value-added role of venture capital and reveals the governance effect of venture capital on corporate fraud. Besides, it provides the theoretical evidence for capitals to better serve the real economy.

## 1. Introduction

In recent years, with the rapid development of the global venture capital industry, relevant research has sprung up. The value-added role of venture capital as an “active investor” is widely recognized in the study of start-ups. Related scholars Chemmanur and others found through research that the enterprises backed by corporate venture capital (CVC) are higher in risk than those backed by independent venture capital (IVC), but the returns are lower; however, measured by patents, an in-depth study of the innovation capabilities of companies found that companies backed by corporate venture capital (CVC) were more capable of innovation, for two possible reasons–failure tolerance and industry knowledge. Alvarez and Dushnitsky used the inherent dichotomy of independent venture capital and corporate investors to study the innovation results of venture capital and found that, in terms of innovation output rate, corporate venture capital is higher than the enterprises backed by independent venture capital. Starting from the medical and health industry, Lehoux et al., when exploring the impact of venture capital on medical technology innovation, found that although there are differences in values and investment objectives of investors, after venture capital enters the medical field, it can promote technology developers and carry out exploration and research on new technologies and develop new medical technologies, thereby enhancing the innovation capability in this field. However, there is currently little research on the subsequent impact of venture capital on firms. The reason for this is the inherent cognitive assumption that VCs will quickly withdraw their investments after the IPO of the invested company and no longer play the role of the investor. However, due to the equity lock-up period, agreement and other reasons, the shares held by venture capital will be restricted from circulation within a certain period of time after the invested company is listed. In addition, when a listed company has good development prospects, venture capital will gradually withdraw its investment from the invested company at an appropriate time in order to maximize the return on investment and obtain sustainable equity income. As mentioned earlier, from the listing of Winner Technology in 2017 to the latest shareholding reduction cycle in January 2022, Sequoia Juye has always been the major shareholder of Winner Technology with a shareholding ratio of more than 5%. Therefore, it is of significance to study the responsibilities of Sequoia Juye as a major shareholder. Based on the above discussion, the research on the supervision and inhibition of VC on corporate fraud is conducted from the perspective of corporate fraud, helping to fully understand the value-added role of VC and expanding the research scope of VC governance [1–10].

Therefore, the data of China's A-share companies from 2010 to 2019 is selected to study the impact of VC shareholding on corporate fraud, to verify the governance role of VC shareholding on companies. According to the research results, the VC-backed companies have a lower probability and frequency of fraud. The result is still valid after endogenous tests such as one period lag, propensity score matching (PSM) and Heckman two-step methods. Through the mechanism test, it is found that VC shareholding has a more significant impact on the fraud probability and fraud frequency of companies with poor internal corporate governance and poor external information environment. Further research found that VC shareholding mainly plays a governance role in the company operation fraud and has no obvious impact on information disclosure and TMT fraud. In the study of nonstate-owned enterprises, VC has a more significant inhibitory effect on the probability and frequency of fraud.

The possible marginal contributions of this paper are as follows. Firstly, this paper enriches the relevant literature on the factors affecting corporate fraud. Most existing studies focused on the effect of motivations and internal and external influence mechanisms on fraud. However, few studies exist on the effect of VC inhibiting corporate fraud. Secondly, this paper enriches the relevant research on the supervision and governance roles of VC. Existing studies on the supervision role of VC often focused on supervising the earnings management of senior executives and phased investment to supervise the development of the company. This study applies the supervision role of VC in the research on various frauds of companies for the first time, enriching the research results on the supervision role of VC.

## 2. Related Technical Methods

**Figure 1:**
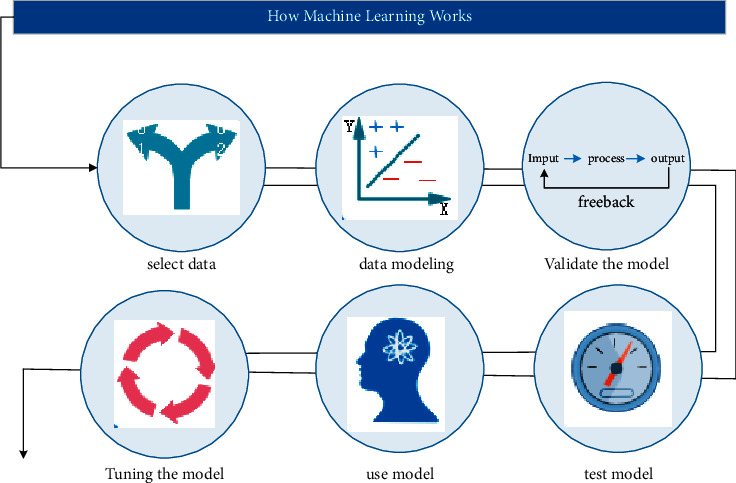
How machine learning works.

Machine learning is all about exploring and developing a set of algorithms that enable computers to learn and model all kinds of data on their own and make predictions using established models and new inputs without explicit instructions from the outside. It has been widely used in various branches of artificial intelligence, such as intelligent customer service in the financial field, robo-advising, behavior recognition, natural language understanding, and risk prediction. Common machine learning algorithms include classification algorithms, regression algorithms, and aggregation algorithms. The working principle of machine learning is simply to substitute specific data samples into the machine learning algorithm for verification and testing. The model is repeatedly tuned, and then the process of application promotion is carried out. How machine learning works is shown in Figure 1 [11–15].

The current mainstream machine learning models are as follows:

1. Classification. Common classification algorithms include support vector machine (SVM) and logistic regression. SVM can quickly classify sample data by discovering the boundaries of separating classes with the widest possible edges. Logistic regression is a process of computing binomial and polynomial classification functions for linearly separable data sources.
2. Regression. A simple definition of regression analysis is the technique of predicting a dependent variable (*y*) based on one or more independent variables (*x*). Regression analysis is usually used for some business decisions, and the common application scenarios are as follows: 6ne is to explain some incomprehensible things. The second is to forecast important business trends. The third is to choose a different option. Linear regression is the most commonly used regression technique. The purpose of linear regression is to find the line of best fit, known as the regression line through the points.
3. Clustering. The basic principle of clustering analysis can be summarized as follows: the aggregation of similar (homogeneous) individuals; that is, “things cluster together, and people are divided into groups.” If you find this class, you can find the similarity of these objects (clusters). Calculations are performed through certain rules, and objects with similarities are aggregated. Common clustering algorithms include clustering of samples, that is, *K*-Means clustering. The main clustering type is to find the proximity of distance, clustering of variables, that is, unified clustering; the key is to find correlations. Proximity. *K*-Means clustering is currently widely used.

Based on machine learning, this paper monitors and experiments venture capital and corporate fraud.

## 3. Hypotheses Development

For companies, VC shareholding affects the motivation of committing fraud by the companies from two aspects. First, suppose that the cost of committing the fraud by the listed company is *c* *=* *p* × *F*, where *p* is the probability of the inspection of the corporate fraud, and *F* is the loss of the company caused by the disclosure of the fraud, including the fine imposed by the regulator and the decline in stock price arising therefrom. The decline in stock price will deal a huge blow to both companies and investors, so it is paid more attention to by the company. In terms of the inspection probability (*p*), as a kind of financial intermediary or institutional investor, VC invests in the companies, often causing more analysts to track and analyze, and thus promoting the company to disclose more information to reduce the degree of information asymmetry [14], so as to improve the information transparency of the company. As “active investors,” VC has rich industry experience and professional analysts and advanced tools, so that it has inherent advantages in obtaining and analyzing information [14]. Therefore, it can be seen that the investment of VC will improve the information transparency of companies and thus improve the probability (*p*) of disclosing the fraud committed by the companies [16–20].

For the company's losses caused by current disclosure fraud (*f*), VC shareholding may produce information spillover effect in some way, which will affect other investors and accelerate the dissemination of negative information of the company. In addition, venture capital holdings have attracted more analysts' attention, which will also accelerate the dissemination of negative information and trigger stock price fluctuations. Therefore, considering the above formula, the possibility that VC holding shares of the company increases the fraud cost of the company does exist (*C* = *P* × *F*). In conclusion, based on the above analysis, this chapter puts forward the following assumptions to be verified based on the above research results:

  H1a: venture backed companies are less likely to commit fraud than nonventure backed companies.
  H1b: compared with nonventure capital backed companies, venture capital backed companies have a lower frequency of fraud.

## 4. Data, Sample Selection, and Variables

### 4.1. Data and Sample

This paper takes the data of all shared companies from 2010 to 2019 as the initial sample, and the relevant data of a company are public data with high authenticity. Through the screening and selection of samples, the details are as follows: (1) eliminate the sample of companies in the finance industry; (2) eliminate ST and *∗*ST samples in abnormal business operation; (3) eliminate the variables and samples with missing values; (4) Winsorize all continuous variables to eliminate the extreme values. This paper mainly studies the variable, and the data on fraud comes from the fraud subdatabase of the China Stock Market and Accounting Research Database (CSMAR). The company's financial data comes from other subdatabases of CSMAR. The relevant characteristic data such as the name of the VC and the number of investment events are all from the CVSource database. As an open database, CVSource database has certain authority and representativeness.

### 4.2. Variable Definition

#### 4.2.1. Dependent Variable

The probability of fraud (Fraud_*i*,*t*+1_): Fraud_*i*,*t*+1_ measures whether the listed company commits fraud, and the data on fraud is cross-sectional data arranged by the fraud events in the subdatabase of the China Stock Market and Accounting Research Database (CSMAR). The data is reorganized into company-year panel data according to the information under the index of “fraud year” because the date of corporate fraud may not be in the same year as the date of fraud audit. If the listed company *i* commits fraud in the year *t* + 1, the Fraud_*i*,*t*+1_ value is 1; otherwise, it is 0. The variable is lagged one period here to alleviate the reverse causality problem to a certain extent. The Probit model is constructed to demonstrate hypothesis H1a, that is, whether VC shareholding effectively reduces the probability of corporate fraud [21].

The frequency of fraud (Freq_*i*,*t*+1_): Freq_*i*,*t*+1_ is used to measure the frequency of fraud committed by the listed company *i* in the year *t* + 1. The variable is lagged one period here to alleviate the reverse causality problem to a certain extent. Poisson model is constructed to Hypothesis H1b, that is, whether VC shareholding reduces the frequency of corporate fraud.

#### 4.2.2. Independent Variable

VC_*i,t*_ is the main research object in this paper. Whether there is a VC among the top ten shareholders of companies is considered the main independent variable. VC_*i,t*_ is a dummy variable and is set to 1 when there is a VC among the top ten shareholders of companies; otherwise, it is 0. The identification process of VC financing is as follows: firstly, confirm VC institutions according to the classification of shareholders of companies in the China Securities Regulatory Commission (CSMAR). Secondly, match the name of VC institutions with their characteristics obtained from the CVSource database by Stata. Finally, the matching result between the name of company shareholders and the full name of VCs is manually confirmed by referring to previous practices [10, 22]. The sample is finally determined through the above steps.

#### 4.2.3. Control Variables

In the selection of control variables, based on the current research edge at home and abroad, this paper selects the following control variables to control other factors: audit quality (AUDIT), company size (scale), property right nature (state), independent director ratio (ind), board size (board size), debt asset ratio (Lev), company years (age), Revenue growth rate (growth), and industry confidence (tqmed). The variables involved in this model and their definitions are shown in Table 1.

## 5. Empirical Tests and Results

### 5.1. Empirical Model

To test hypothesis H1, that is, the impact of VC shareholding on corporate fraud, the model is constructed as follows:

(1) $$\begin{array}{c} Y_{i,t+1}=\beta_{0}+\beta_{1}\times VC_{i,t}+\beta_{2}\times {\text{Controls}}_{i,t}+{\text{Year}}_{i,t}+{\text{Industry}}_{i,t}+\varepsilon_{i,t}. \end{array}$$

where the dependent variable is the fraud status of the companies next year, and the reason to use one period lag is to alleviate the endogeneity problem such as reverse causality. *Y*_*i*,*t*+1_ is expressed by Fraud_*i*,*t*+1_ and Freq_*i*,*t*+1_ respectively in this paper. In the regression process, the Probit model is used for the dummy variable Fraud; the Poisson model is used for the counting variable Freq. The main independent variable is whether there is VC (*VC*_*i*,*t*_) among the top ten shareholders of companies; Control_*i,t*_ is a series of control variables; in addition, the model also controls the annual and industry fixed effects. The regression coefficient *β* reflects the impact of the core independent variable “VC holdings” on corporate fraud, and it is the empirical result this paper focuses on. If VCs have a governance effect on companies, the fraud probability and fraud frequency of VC-backed companies are significantly lower than those of non-VC-backed companies; that is, *β* should be significantly negative [23].

### 5.2. Summary Statistics

In this paper, the summary statistics of all variables are shown in Table 2. The mean value of corporate fraud (Fraud_*i*,*t*+1_) is 0.203, indicating that about 20.3% of companies commit fraud in all sample observations. The mean value of corporate fraud frequency (Freq_*i*,*t*+1_) is 0.314, the standard deviation is 0.735, the minimum value is 0, and the maximum value is 4, indicating that the average frequency of corporate fraud is about 0.3 in the annual observations, and the fluctuation range around the mean value is large. In terms of independent variables, the mean value of VC (*VC*_*i*,*t*_) is 0.266, indicating that the average proportion of companies with a VC shareholder among the top ten shareholders during the sample period is about 26.6% [24].

In addition, the summary statistics of the control variables show that the mean value of whether any of the big 4 accounting firms are hired is 0.040, indicating that about 4% of the companies hire the big 4 accounting firms in annual observations of all companies. For corporate governance factors, the mean value of the CEO duality is 0.729; that is, the probability of the Chair of the Board concurrently serving as the CEO is up to 72.3% in the sample range of this study, indicating that the Chair of the Board concurrently serving as the CEO is a quite common phenomenon in the current situation of listed corporate governance in China. The mean value of the shareholding ratio of the largest shareholder (Top1) is 0.353, the minimum value is 0.09, and the maximum value is 0.75, indicating that there is a relatively large gap in the shareholding ratio of the largest shareholder between China's companies, and the shareholding ratio is generally high. The mean value of the shareholding ratio is consistent with the existing research conclusions.

### 5.3. Basic Results

Table 3 shows the empirical results of the impact of VC shareholding (*VC*_*i*,*t*_)) on violations of companies. In the regression, the variables that may affect corporate fraud are controlled, as well as the fixed effects of year and industry. Columns (1) and (2) show the results of the impact of VC shareholding (*VC*_*i*,*t*_) on corporate fraud (Fraud_*i*,*t*+1_). Columns (3) and (4) show the results of the impact of VC shareholding (*VC*_*i*,*t*_) on the probability (Freq_*i*,*t*+1_) of corporate fraud. According to column (2) of Table 3, the estimated coefficient of the impact of VC shareholding (*VC*_*i*,*t*_) on corporate fraud (Fraud_*i*,*t*+1_) is significantly negative at the significance level of 5% after the possible factors influencing the corporate fraud are controlled except for VC shareholding, indicating that the fraud probability of the VC-backed companies will significantly decrease in the next year. According to column (4) of Table 3, the estimated coefficient of the impact of VC shareholding (*VC*_*i*,*t*_) on the fraud probability (Freq_*i*,*t*+1_) of companies is significantly negative at the significance level of 1% after the possible factors influencing the corporate fraud are controlled except for VC shareholding, indicating that VC shareholding effectively will reduce the fraud frequency of the companies in the next year. To sum up, the hypothesis in this paper is confirmed; that is, VC significantly inhibits the occurrence of corporate fraud [25].

According to the results of control variables, the coefficient of audit quality (Audit) is significantly negative but not significant, indicating that independent high-quality external audit and supervision can effectively reduce the probability and frequency of corporate fraud and supervise and restrain the occurrence of corporate fraud. The coefficient of return on assets (ROA) is significantly negative possibly because there is less incentive to obtain illegitimate incomes by fraud when the return on assets is more. The coefficient of the board size (Bdsize) is significantly positive, indicating that larger board size is not equivalent to a higher board efficiency. The larger the size of the board of directors, the more likely there are differences in interests and failure of supervision. The coefficient of CEO duality (Dual) is negative, indicating that the higher the CEO duality, the less the frequency of fraud. The possible reason is that the person bears more responsibilities and receives more severe punishment when holding the positions of the Chair of Board and CEO concurrently, inhibiting the persons' motivation to commit fraud more effectively.

### 5.4. Mechanism Test

In this section, the internal and external governance environment mechanism of companies is tested to clarify whether VC affects corporate fraud by participating in the governance and supervision of the companies.

#### 5.4.1. Internal Governance Environment

The internal governance environment affects the efficiency of supervisors. VC shareholding plays a positive role in the supervision of companies by means of participating in the general meeting of shareholders [12] and assigning directors to the investee company. The role of supervision of VC varies with internal governance environments. When the internal governance environment of the company is poor, the management and large shareholders have more motivations and opportunities to act in their own self-interest, so the fraud frequency of the companies is higher. In such a case, the impact of VC shareholding on the governance of corporate fraud is more significant. On the contrary, if a company has a good original internal governance environment and a good supervision mechanism to restrict the self-interest motivation of insiders, then VC institutions, providing supplementary supervision for the internal governance of the company, only have small marginal benefits [22].

Furthermore, the benefit brought by active participation in supervision is far greater than the cost, so venture capitalists are motivated to implement active supervision. Active supervision helps improve the intrinsic value of companies and finally improves the exit return of VC, while negative supervision increases the cost: (1) it directly affects the stock price of companies and reduces the exit return of VC; (2) it indirectly affects the reputation accumulation of VC industry and then affects the subsequent financing and investment of venture capitalists. Therefore, venture capitalists are willing and motivated to supervise companies (Tam, 2010). Based on the above reasoning, the following inference is proposed:

**Inference 1 .**

The worse the internal governance environment, the more significant the impact of VC on corporate fraud.

The fund occupation is used as the proxy variable of the internal governance environment in this paper. Fund occupation is an important means for corporate insiders to seek private interests [11], so fund occupation is selected as the proxy variable of the internal governance environment. It is calculated by the following formula: Occupancy = (other receivables − other payables)/total assets. The greater the occupancy value, the more the occupied funds of the listed company, and the worse the internal governance environment of the company. In this section, the average value of the capital occupation level in the industry in the previous year is used as the grouped variable, and the sample is divided into two groups, namely, the group with good internal governance (Occupancy -L) and the group with poor internal governance (Occupancy –H).  Table 4 shows the results of grouped regression.

According to the results in columns (1) and (2) of Table 4, the coefficient of the impact of VC shareholding (VC_*i,t*_) on the fraud probability (Fraud_*i*,*t*+1_) of companies is negative in the subsamples whether the internal environment is good or bad, but it is significantly negative in the subsamples with the poor internal environment (Occupancy-l). The results in columns (3) and (4) of Table 4 show that the coefficient of the impact of VC shareholding (VC_*i,t*_) on the fraud frequency of companies (Freq_*i*,*t*+1_) is also significantly negative in the group with the poor internal environment (Occupancy-l). The above results confirm the abovementioned Inference 1; that is, the worse the internal governance environment of the company, the more significant the impact of VC on fraud.

#### 5.4.2. External Governance Environment

According to the modern information-based financial intermediary theory, information production is the root cause for the existence of financial intermediary [21]. The role of certification of VC shareholders on enterprises is reflected in its shareholding, conveying a “good profit” signal to the market. VC shareholding will release certification signals to the market, which will drive the stock trading volume of companies to rise. Existing studies have shown that stock liquidity is also an indirect governance mechanism [8, 20]. VCs are special financial investment institutions, so their investment projects are more likely to attract more attention from analysts, and more internal information of the investee company is transmitted to the market in the form of analyst reports, thus improving the information transparency of the enterprise. In addition, as a professional investment institution, VC can effectively identify the impact of various information on the company's stock price and development prospects. Therefore, the shareholding status of VC implies the trend of the company's future development [14].

The impact of the external information environment on the relationship between VC and corporate fraud is as follows: the original information transparency of companies will affect the relationship between the VC shareholding and corporate fraud. When the transparency of the company's external information environment is low, investors lack approaches to acquiring the internal information of companies, resulting in a huge information gap. Under such circumstances, it is less likely to be found by the outside world even if the company commits fraud, or the adverse impact arising from corporate fraud is relatively small even if corporate fraud is found in an audit. Therefore, there may be more serious fraud in such companies, the venture capitals are provided with good opportunities to play the role of governance, and the supervision role of VC shareholding in corporate governance will be more significant. On the contrary, if the external information transparency of the company is high, and the self-interest motivation of insiders can be effectively restrained, the governance space for VC is relatively small, so as an alternative to the external governance mechanism, the supervision role of VC will be inhibited to a certain extent. As an “active investor,” the VC-backed companies will attract more attention. For example, more analysts will disclose more enterprise information to the market, thus reducing the asymmetry between internal and external information [14]. The higher the information transparency, the greater the probability of fraud being found. The following inference is proposed based on the above analysis:

**Inference 2 .**

The worse the external information environment the company is in, the more significant the impact of VC on corporate fraud is.

According to the existing studies, the number of analysts tracking the companies is selected as the proxy variable of the external information environment. Firstly, the samples are grouped into better information environment (Analyst-H) and worse information environment (Analyst-L) according to the industry median of the number of analysts in the previous year. Then, grouped regression is performed, and the regression results are shown in Table 5. The results in columns (1) and (2) show that the coefficient of the impact of VC shareholding on the probability of corporate fraud (Fraud_*i*,*t*+1_) is significantly negative at the 10% level in the subsamples with poor external information environment (Analyst-L). The results in columns (3) and (4) show that the coefficient of the impact of VC shareholding on the frequency of corporate fraud (Freq_*i*,*t*+1_) is significantly negative at the level of 1% in the subsamples with poor external information environment (Analyst-L). The above-mentioned Inference 2 is confirmed through the above results; that is, the worse the external information environment of the company, the more significant the impact of VC on corporate fraud.

## 6. Endogeneity and Robustness Test

### 6.1. Endogeneity Test

The variable such as the industry and year is controlled, and the corresponding control variables are selected according to different types, but there are still inevitable endogeneity problems. There may be the following endogenous problems in this paper: the first is reverse causality. VC shareholding can improve the governance and efficiency of companies and reduce the probability of corporate fraud through supervision and governance. Furthermore, venture investors do not randomly choose companies for investment but tend to choose enterprises with good future development prospects, good company operation, good corporate governance, and fewer frauds. The second is selection bias, namely, self-selection bias and sample selection bias. In terms of self-selection bias, due to the “selection effect,” VC may choose enterprises with better corporate governance effect, thus resulting in the wrong identification of governance effects of VC. Furthermore, whether enterprises choose VC is self-selection and will be affected by various factors. In terms of sample selection bias, there may be some differences between VC-backed companies and non-VC-backed companies, leading to the differences in fraud between an experimental group and a control group. Endogeneity is tested as follows:

#### 6.1.1. Reverse Causality

By referring to the research methods of the VC and other control variables are treated with a lag period, and then the fraud data of the company (i) in the current period (*t*) and the independent variables lagged one period are regressed. See Table 6 for the results.

The results in column (1) of Table 6 show that the coefficient of the impact of VC shareholding on the probability of corporate fraud (Fraud_*i,t*_) is significantly negative at the significance level of 10%. The results in column (2) show that the coefficient of the impact of VC shareholding on the frequency of corporate fraud (Freq_*i,t*_) is significantly negative at the significance level of 1%. To sum up, after considering the possible endogeneity problems such as reverse causality, the main test conclusion is still stable; that is, VC shareholding can effectively reduce the probability and the frequency of corporate fraud.

#### 6.1.2. Self-Selection Problem

In this section, Heckman two-step method is used to handle the possible sample self-selection problems. The specific steps are as follows: the first step is that Probit regression is used to model the relationship between the dummy variable VC as the dependent variable and two relative exogenous variables (exclusion constraints) affecting the selection of investee companies by VC, and the inverse Mills ratio (IMR) is calculated. Exclusion constraints are strongly correlated with independent variables and will not directly affect the dependent variables. Based on the existing practices, whether the selected company is in the area with active VC investments and the period of rapid development of VC is used as exclusion constraints. For the identification of the areas with active VC investments, according to the Venture Capital Development in China, companies headquartered in Beijing, Jiangsu, and Guangdong are identified as the companies in areas with active VC investments and are set as a dummy variable Z1; for the identification of the development period of VC industry, China's venture capital industry began to develop rapidly after the “No. 1 Proposal” of the Chinese People's Political Consultative Conference (CPPCC) in 1998, so the value of the companies after 1998 is set to 1 in this paper, and such companies are easier to get the VC support. In the second step, the inverse Mills ratio (IMR) is used in the regression and regressed with VC and control variables. If the IMR coefficient is not significant, and the coefficient of VC is still significantly negative, it indicates that the model is still considered to be robust after the sample self-selection bias is taken into consideration. See Table 7 for specific estimation results. The results show that the impact of VC on the probability and frequency of corporate fraud is still significantly negative in the second step, indicating that the basic test in this paper is still robust after the self-selection problem is considered.

#### 6.1.3. Sample Selection Bias

The above results may be affected by the sample selection bias, so there is a “selection effect” of VC on the investee company. The difference in fraud tendency between the treatment group and the control group of VC may be caused by inherent differences between the two groups of samples. To alleviate this problem, the propensity score matching method (PSM) is used to select the appropriate control group for VC enterprises to reduce the systematic difference between enterprises with and without VC investments. The specific matching methods are as follows: firstly, the years from 2010 to 2019 are the sample period, the control group as the enterprises not selected by VC during the sample period, and the control variables in the benchmark model and the exclusion constraints in the Heckman selection model are used as covariates to perform Logit regression and the propensity matching score is obtained. Then, the nearest neighbor 1 : 1 and radius matching method is used to find an appropriate control group for the experimental group, and the caliper radius is selected to be 0.01.

The balance test results in Table 8 can reflect the matching effect of PSM. It can be seen from the test results of Table 8 that other covariates showed significant differences before matching except ROA and Dual, but the difference between covariates disappeared after PSM, indicating that PSM reduced the systematic difference between the experimental group and the control group and the matching effect of PSM was better. Additionally, from the perspective of deviation percentage, the standardization difference of covariates is less than 10%, and the standardization deviation of most variables is significantly reduced compared with that before matching. In conclusion, the matching method satisfies the equilibrium hypothesis well.

The main test for matched samples by PSM is regressed. The regression results for the matched samples by PSM are shown in Table 9. Columns (1) and (2) are the regression results by using nearest neighbor 1 : 1 matching. Columns (3) and (4) are the regression results by using nearest neighbor 1 : 2 matching. The results in Table 9 show that the coefficient of the impact of VC shareholding on the probability of corporate fraud (Fraud_*i,t+1*_) and the frequency of corporate fraud (Freq_*i,t+1*_) is significantly negative whether nearest neighbor 1 : 1 matching or nearest neighbor 1 : 2 matching is used; that is, the conclusion of the basic test is still robust after the possible endogeneity problems are solved by PSM.

### 6.2. Robustness Test

#### 6.2.1. Replace Method

To investigate whether the impact of VC shareholding on corporate fraud depends on the estimation method of the model, the Tobit model is first used to retest the impact of VC shareholding on the probability of corporate fraud. Subsequently, negative binomial regression is used to test the relationship between VC shareholding and the frequency of corporate fraud. The fraud frequency for only the company that commits fraud is non-zero, the independent variable changes from zero to non-zero, and then excessive dispersion may appear, so negative binomial regression is used instead of the abovementioned Poisson model to test robustness to avoid the impact of excessive dispersion on the research. The results of the robustness test are shown in Table 10. The results in column (1) show that the coefficient of the impact of VC shareholding on the probability of corporate fraud is still significantly negative after the replacement of the Tobit model; the results in column (2) show that the coefficient of the impact of VC shareholding on the frequency of corporate fraud is still significantly negative after the use of negative binomial regression. To sum up, the basic test results are still robust.

#### 6.2.2. Sample Transformation and Winsorization Change

By referring to previous studies (Zou et al., 2019), the missing values are not eliminated, and winsorization is not used for samples. The basic test was regressed again with new samples, and the results are shown in Table 11. Without eliminating the missing value and without winsorization, the coefficient of the impact of VC shareholding (VC_*i,t*_) on the probability of corporate fraud (Fraud_*i,t+1*_) is significantly negative at the significance level of 5%. The coefficient of the impact of the frequency of corporate fraud (Freq_*i,t+1*_) is significantly negative at the significance level of 1%. The above results show that the conclusion of the basic test is still robust in this paper and will not be changed by changing the time range and winsorization of samples.

## 7. Conclusions

This paper selects all A-share companies from 2010 to 2019 as samples to study the impact of VC Shareholding on corporate fraud. According to the research results, firstly, the basic test confirms that, compared with non-VC supported companies, the possibility and frequency of fraud of VC supported companies are lower, and the influence coefficient of VC shareholding (VC_*i*,*t*_) on the probability of corporate fraud (fraud_*i*,*t*+1_) is significantly negative when the significance level is 5%. When the significance level is 1%, the influence coefficient of corporate fraud frequency (freq_*i*,*t*+1_) is significantly negative. The above conclusions show that VC shareholding strengthens the supervision for companies and lowers the probability of the market punishment due to fraud by playing the role of supervision and governance, so the agency cost is reduced, and the governance mechanism of investee companies is improved. Secondly, through the mechanism test, it is found that the method of VC shareholding to reduce corporate fraud is to curb its fraud by improving the company's information environment. In terms of the internal governance environment, the coefficient of the impact of VC on the companies with a worse internal information environment is more significant; in terms of the external information environment, when the external information environment is poor, VC has a more significant inhibitory effect on the corporate fraud. Thirdly, the endogeneity and robustness are tested. To alleviate the possible endogeneity problems such as reverse causality, sample selection bias, and self-selection bias, this paper has confirmed that the conclusion is still robust after controlling the endogeneity problem through the methods of one period lag, Heckman two-step test, and propensity score matching (PSM). In addition, the robustness is tested by changing the estimation model and using the samples without eliminating missing values and without winsorization, and the results are still valid. Finally, further research found that VC shareholding plays an effective role in the governance of the company's information disclosure fraud and operation fraud but not a significant role in the governance of TMT fraud. Compared with state-owned enterprises, VC has a better inhibitory effect on the frequency of corporate fraud in nonstate-owned enterprises through its supervision and governance role.

## Figures and Tables

**Table 1:** Variable definitions.

Variable name	Symbol	Description
*Main variable*		
Corporate fraud	Fraud_*i*,*t*+1_	The dummy variable, is set to 1 if there is corporate fraud, otherwise it is 0
Fraud frequency	Freq_*i*,*t*+1_	Count variable, corporate fraud frequency
Venture capital	*VC*_*i*,*t*_	The dummy variable, is set to 1 if the VC holds the shares of companies in the current year, otherwise it is 0

*Control variable*		
Audit quality	*Audit*	The dummy variable, is set to 1 if audit institutions are big 4 accounting firms, otherwise it is 0
Company size	*Size*	Natural logarithm of total assets
Performance variable	*Roa*	Return on assets (ROA)
Board size	*Bdsize*	The logarithm of the number of directors by the end of the year
CEO duality	*Dual*	The value is set to 1 if the roles of the CEO and the chair of the board are combined, otherwise it is 0
Years of being companies	*Age*	The logarithm of years of being companies
Industry confidence	*Tqmed*	Median of TobinQ of all companies in the same industry by the end of the year
Industry dummy variable	*Industry*	Classified according to the industry classification standard of the China securities regulatory commission (CSRC) in 2012
Year dummy variable	*Year*	Year dummy variable

**Table 2:** Descriptive statistics.

Variable	Sample size	Mean value	Standard deviation	Minimum value	Maximum value
Fraud	16347	0.203	0.402	0.000	1.000
Freq	16347	0.314	0.735	0.000	4.000
VC	16347	0.266	0.442	0.000	1.000
Audit	16347	0.040	0.195	0.000	1.000
Age	16347	1.886	0.926	0.000	3.178
Cash	16347	0.438	0.848	-2.355	3.748
Size	16347	22.265	1.199	20.085	25.884
ROA	16347	0.047	0.051	-0.155	0.197
Dual	16347	0.729	0.444	0.000	1.000
Top1	16347	0.353	0.150	0.090	0.750
BdSize	16347	2.143	0.195	1.609	2.708
Tqmed	16347	1.756	0.528	0.996	3.648

**Table 3:** VC shareholding and corporate fraud.

	(1)	(2)	(3)	(4)
	Probit		Poisson	
	Fraud_*t*+1_	Fraud_*t*+1_	Freq_*t*+1_	Freq_*t*+1_
VC	−0.0560^*∗*^	−0.0642^*∗∗*^	−0.134^*∗∗∗*^	−0.149^*∗∗∗*^
	(−1.80)	(−2.03)	(−3.44)	(−3.80)
Age		−0.0333^*∗*^		−0.0484^*∗∗*^
		(−1.87)		(−2.18)
Size		0.00923		0.0534^*∗∗∗*^
		(0.59)		(2.82)
ROA		−3.615^*∗∗∗*^		−6.101^*∗∗∗*^
		(−11.13)		(−16.68)
Dual		−0.0912^*∗∗∗*^		−0.178^*∗∗∗*^
		(−2.93)		(−4.77)
Audit		−0.243^*∗∗∗*^		−0.397^*∗∗∗*^
		(−3.10)		(−3.72)
BdSize		0.156^*∗∗*^		0.171^*∗*^
		(2.06)		(1.87)
Tqmed		−0.0552		−0.146^*∗*^
		(−0.89)		(−1.90)
Cash		−0.0795^*∗∗∗*^		−0.133^*∗∗∗*^
		(−4.42)		(−5.98)
Constant	−0.275	−0.301	−0.303^*∗*^	−0.869^*∗*^
	(−1.55)	(−0.73)	(−1.86)	(−1.82)
Observations	12003	12003	12047	12047
Year&Industry	Yes	Yes	Yes	Yes
Pseudo *R*^2^	0.018	0.036	0.024	0.047
AIC	12024.7	11819.1	18581.6	18156.5
Log likelihood	−5931.3	−5820.6	−9201.8	−8981.2

^*∗*^ *p* < 0.10, ^*∗∗*^*p* < 0.05, and ^*∗∗∗*^*p* < 0.01.

**Table 4:** Internal governance environment.

	(1)	(2)	(3)	(4)
	Fraud_*t*+1_		Freq_*t*+1_	
	Occupy-H	Occupy-L	Occupy-H	Occupy-L
VC	−0.043	−0.089^*∗*^	−0.058	−0.277^*∗∗∗*^
	(−1.10)	(−1.66)	(−1.21)	(−4.02)
Age	−0.040^*∗*^	−0.022	−0.070^*∗∗*^	0.001
	(−1.76)	(−0.72)	(−2.48)	(0.02)
Size	0.018	0.003	0.074^*∗∗∗*^	0.039
	(0.87)	(0.14)	(2.99)	(1.27)
ROA	−4.405^*∗∗∗*^	−2.633^*∗∗∗*^	−6.711^*∗∗∗*^	−5.177^*∗∗∗*^
	(−10.30)	(−5.15)	(−13.85)	(−9.11)
Dual	−0.037	−0.186^*∗∗∗*^	−0.070	−0.338^*∗∗∗*^
	(−0.93)	(−3.56)	(−1.47)	(−5.55)
Audit	−0.311^*∗∗∗*^	−0.177	−0.435^*∗∗∗*^	−0.346^*∗∗*^
	(−2.68)	(−1.61)	(−2.80)	(−2.29)
BdSize	0.062	0.286^*∗∗*^	−0.040	0.433^*∗∗∗*^
	(0.64)	(2.31)	(−0.34)	(2.88)
Tqmed	−0.080	−0.031	−0.207^*∗∗*^	−0.065
	(−0.99)	(−0.30)	(−2.06)	(−0.54)
Cash	−0.072^*∗∗∗*^	−0.098^*∗∗∗*^	−0.148^*∗∗∗*^	−0.116^*∗∗∗*^
	(−3.07)	(−3.37)	(−5.12)	(−3.24)
Constant	−0.096	−0.619	−0.498	−1.578^*∗∗*^
	(−0.18)	(−0.93)	(−0.82)	(−1.99)
Observations	7478	4471	7508	4539
Year&Industry	Yes	Yes	Yes	Yes
Pseudo *R*^2^	0.040	0.049	0.049	0.067
AIC	7423.775	4443.219	11370.060	6796.922
Log likelihood	−3622.888	−2139.610	−5588.030	−3307.461

^*∗*^ *p* < 0.10, ^*∗∗*^*p* < 0.05, and ^*∗∗∗*^*p* < 0.01.

**Table 5:** External governance environment.

	(1)	(2)	(3)	(4)
	Fraud_*t*+1_		Freq_*t*+1_	
	Analyst-H	Analyst-L	Analyst-H	Analyst-L
VC	−0.047	−0.077^*∗*^	−0.122	−0.158^*∗∗∗*^
	(−0.96)	(−1.83)	(−1.57)	(−3.09)
Age	−0.073^*∗∗*^	−0.030	−0.099^*∗∗∗*^	−0.038
	(−2.47)	(−1.27)	(−2.60)	(−1.36)
Size	−0.009	0.063^*∗∗∗*^	0.015	0.129^*∗∗∗*^
	(−0.33)	(2.85)	(0.45)	(5.06)
ROA	−2.606^*∗∗∗*^	−3.853^*∗∗∗*^	−5.811^*∗∗∗*^	−5.759^*∗∗∗*^
	(−4.98)	(−8.33)	(−9.20)	(−11.64)
Dual	−0.077	−0.095^*∗∗*^	−0.235^*∗∗∗*^	−0.135^*∗∗∗*^
	(−1.56)	(−2.30)	(−3.94)	(−2.79)
Audit	−0.237^*∗∗*^	−0.249^*∗∗*^	−0.434^*∗∗∗*^	−0.365^*∗∗*^
	(−2.18)	(−2.11)	(−2.80)	(−2.42)
BdSize	0.208^*∗*^	0.129	0.146	0.184
	(1.74)	(1.29)	(0.99)	(1.56)
Tqmed	0.009	−0.104	−0.038	−0.193^*∗*^
	(0.10)	(−1.24)	(−0.32)	(−1.89)
Cash	−0.099^*∗∗∗*^	−0.064^*∗∗∗*^	−0.170^*∗∗∗*^	−0.105^*∗∗∗*^
	(−3.59)	(−2.61)	(−4.74)	(−3.57)
Constant	−0.380	−1.156^*∗∗*^	−0.372	−2.368^*∗∗∗*^
	(−0.57)	(−2.05)	(−0.45)	(−3.78)
AME			−0.0334^*∗∗*^	−0.0555^*∗∗∗*^
			(−1.96)	(−3.08)
Observations	5374	6555	5459	6588
Year&Industry	Yes	Yes	Yes	Yes
Pseudo *R*^2^	0.054	0.033	0.075	0.044
AIC	4922.074	6913.573	7433.147	10667.752
Log likelihood	−2377.037	−3370.786	−3619.573	−5242.876

^*∗*^ *p* < 0.10, ^*∗∗*^*p* < 0.05, and ^*∗∗∗*^*p* < 0.01.

**Table 6:** Variable lagged one period.

	(1)	(2)
	Fraud_*t*_	Freq_*t*_
L.VC	−0.062^*∗*^	−0.140^*∗∗∗*^
	(−1.95)	(−3.60)
L.Age	−0.035^*∗∗*^	−0.054^*∗∗*^
	(−1.99)	(−2.45)
L.Size	0.011	0.058^*∗∗∗*^
	(0.68)	(3.04)
L.ROA	−3.608^*∗∗∗*^	−6.081^*∗∗∗*^
	(−11.11)	(−16.60)
L.Dual	−0.089^*∗∗∗*^	−0.174^*∗∗∗*^
	(−2.86)	(−4.67)
L.Audit	−0.244^*∗∗∗*^	−0.404^*∗∗∗*^
	(−3.11)	(−3.78)
L.BdSize	0.161^*∗∗*^	0.169^*∗*^
	(2.12)	(1.84)
L.Tqmed	−0.029	−0.088
	(−0.50)	(−1.22)
L.Cash	−0.079^*∗∗∗*^	−0.134^*∗∗∗*^
	(−4.36)	(−6.02)
Constant	−0.400	−1.093^*∗∗*^
	(−0.98)	(−2.32)
Observations	11998	12047
Year and industry	Yes	Yes
Pseudo *R*^2^	0.035	0.046
AIC	11826.115	18162.879
Log likelihood	−5826.057	−8986.439

^*∗*^ *p* < 0.10, ^*∗∗*^*p* < 0.05, and ^*∗∗∗*^*p* < 0.01.

**Table 7:** Heckman two-step estimation result.

	(1)	(2)	(3)
	Step 1	Step 2	
	VC	Fraud_*t*+1_	Freq_*t*+1_
*Z*1	0.040^*∗*^		
	(1.67)		
*Z*2	0.093^*∗∗∗*^		
	(4.00)		
VC		−0.065^*∗∗*^	−0.149^*∗∗∗*^
		(−2.04)	(−3.81)
IMR		−0.062	−0.125
		(−0.16)	(−0.27)
Age		−0.031	−0.045^*∗*^
		(−1.60)	(−1.88)
Size		0.008	0.053^*∗∗∗*^
		(0.53)	(2.77)
ROA		−3.603^*∗∗∗*^	−6.090^*∗∗∗*^
		(−11.09)	(−16.64)
Dual		−0.092^*∗∗∗*^	−0.178^*∗∗∗*^
		(−2.93)	(−4.77)
Audit		−0.242^*∗∗∗*^	−0.397^*∗∗∗*^
		(−3.09)	(−3.71)
BdSize		0.155^*∗∗*^	0.171^*∗*^
		(2.05)	(1.86)
Tqmed		−0.064	−0.153^*∗∗*^
		(−1.02)	(−1.98)
Cash		−0.080^*∗∗∗*^	−0.133^*∗∗∗*^
		(−4.42)	(−5.97)
Constant	−1.228^*∗∗∗*^	−0.165	−0.636
	(−7.24)	(−0.22)	(−0.69)
Observations	16297	11966	12007
Year and industry	Yes	Yes	Yes
Pseudo *R*^2^	0.074	0.035	0.046
AIC	17665.601	11803.346	18132.111
Log likelihood	−8746.800	−5813.673	−8973.055

^*∗*^ *p* < 0.10, ^*∗∗*^*p* < 0.05, and ^*∗∗∗*^*p* < 0.01.

**Table 8:** PSM.

Variable	Unmatched matched	Mean		%bias	% reduct |bias|	*t*-test	*p* > |*t*|
		Treated	Control			*t*	
*Z*1	U	0.3731	0.3473	5.40		3.05	0.002
	M	0.3702	0.3822	−2.50	53.30	−1.16	0.248
*Z*2	U	0.5774	0.5285	9.80		5.55	0.000
	M	0.5749	0.5742	0.10	98.60	0.07	0.948
Age	U	1.9434	1.8646	8.50		4.82	0.000
	M	1.9415	1.9183	2.50	70.60	1.19	0.234
Size	U	22.5030	22.1790	26.40		15.37	0.000
	M	22.4870	22.4910	−0.30	99.00	−0.12	0.903
ROA	U	0.0469	0.0465	0.70		0.41	0.683
	M	0.0468	0.0471	−0.60	17.90	−0.28	0.779
Dual	U	0.7320	0.7279	0.90		0.52	0.604
	M	0.7325	0.7348	−0.50	43.30	−0.24	0.808
Audit	U	0.0552	0.0342	10.20		6.09	0.000
	M	0.0526	0.0556	−1.50	85.70	−0.62	0.536
BdSize	U	2.1602	2.1368	12.00		6.76	0.000
	M	2.1593	2.1583	0.50	95.50	0.25	0.805
Tqmed	U	1.8183	1.7334	15.80		9.11	0.000
	M	1.8137	1.8092	0.80	94.80	0.36	0.717
Cash	U	0.4736	0.4249	5.70		3.25	0.001
	M	0.4652	0.4662	−0.10	97.90	−0.05	0.956

**Table 9:** Regression result based on PSM.

	(1)	(2)	(3)	(4)
	Radius-nearest neighbor (1 : 1) matching		Radius-nearest neighbor (1 : 2) matching	
	Fraud_*t*+1_	Freq_*t*+1_	Fraud_*t*+1_	Freq_*t*+1_
VC	−0.084^*∗*^	−0.152^*∗∗∗*^	−0.076^*∗∗*^	−0.159^*∗∗∗*^
	(−1.92)	(−2.82)	(−2.06)	(−3.52)
Age	−0.058^*∗∗*^	−0.099^*∗∗∗*^	−0.057^*∗∗*^	−0.085^*∗∗∗*^
	(−2.00)	(−2.76)	(−2.42)	(−2.88)
Size	0.025	0.107^*∗∗∗*^	0.015	0.084^*∗∗∗*^
	(1.01)	(3.55)	(0.77)	(3.43)
ROA	−3.562^*∗∗∗*^	−5.654^*∗∗∗*^	−3.566^*∗∗∗*^	−6.267^*∗∗∗*^
	(−6.95)	(−9.24)	(−8.35)	(−12.51)
Dual	−0.048	−0.128^*∗∗*^	−0.043	−0.110^*∗∗*^
	(−0.97)	(−2.17)	(−1.06)	(−2.25)
Audit	−0.305^*∗∗*^	−0.335^*∗∗*^	−0.214^*∗∗*^	−0.234^*∗*^
	(−2.55)	(−2.19)	(−2.22)	(−1.90)
BdSize	0.222^*∗*^	0.196	0.230^*∗∗*^	0.198^*∗*^
	(1.84)	(1.34)	(2.35)	(1.67)
Tqmed	−0.007	−0.023	−0.040	−0.075
	(−0.07)	(−0.18)	(−0.50)	(−0.75)
Cash	−0.081^*∗∗∗*^	−0.139^*∗∗∗*^	−0.067^*∗∗∗*^	−0.120^*∗∗∗*^
	(−2.91)	(−4.01)	(−2.88)	(−4.19)
Constant	−1.055	−2.501^*∗∗∗*^	−0.758	−1.748^*∗∗∗*^
	(−1.56)	(−3.15)	(−1.41)	(−2.79)
Observations	4962	5002	7191	7226
Year&Industry	Yes	Yes	Yes	Yes
Pseudo *R*^2^	0.046	0.054	0.039	0.050
AIC	4919.764	7447.950	7103.497	10869.554
Log likelihood	−2373.882	−3629.975	−3463.748	−5339.777

^*∗*^ *p* < 0.10, ^*∗∗*^*p* < 0.05, and ^*∗∗∗*^*p* < 0.01.

**Table 10:** Replace model.

	(1)	(2)
	Tobit	Negative binomial regression
	Fraud_*t*+1_	Freq_*t*+1_
VC	−0.018^*∗∗*^	−0.132^*∗∗*^
	(−2.09)	(−2.55)
Age	−0.009^*∗*^	−0.041
	(−1.87)	(−1.39)
Size	0.004	0.033
	(0.84)	(1.25)
ROA	−0.999^*∗∗∗*^	−6.259^*∗∗∗*^
	(−11.46)	(−11.75)
Dual	−0.026^*∗∗∗*^	−0.173^*∗∗∗*^
	(−2.98)	(−3.42)
Audit	−0.056^*∗∗∗*^	−0.378^*∗∗∗*^
	(−2.96)	(−2.85)
BdSize	0.037^*∗*^	0.239^*∗*^
	(1.84)	(1.92)
Tqmed	−0.016	−0.123
	(−0.95)	(−1.17)
Cash	−0.020^*∗∗∗*^	−0.134^*∗∗∗*^
	(−4.25)	(−4.51)
Constant	0.375^*∗∗∗*^	−0.662
	(3.32)	(−0.98)
Observations	12047	12047
Year and industry	Yes	Yes
Pseudo *R*^2^	0.037	0.028
AIC	11914.801	16851.091
Log likelihood	−5859.400	−8327.546

^*∗*^ *p* < 0.10, ^*∗∗*^*p* < 0.05, and ^*∗∗∗*^*p* < 0.01.

**Table 11:** Winsorization change without eliminating missing value.

	(1)	(2)
	Probit	Poisson
	Fraud_*t*+1_	Freq_*t*+1_
VC	−0.0483^*∗∗*^	−0.133^*∗∗∗*^
	(−2.01)	(−4.83)
Age	0.0133	0.0762^*∗∗∗*^
	(1.07)	(5.36)
Size	−0.0251^*∗∗*^	−0.0252^*∗∗*^
	(−2.54)	(−2.25)
ROA	−2.653^*∗∗∗*^	−2.152^*∗∗∗*^
	(−17.23)	(−27.42)
Dual	−0.118^*∗∗∗*^	−0.199^*∗∗∗*^
	(−5.05)	(−7.73)
Audit	−0.276^*∗∗∗*^	−0.446^*∗∗∗*^
	(−5.42)	(−6.76)
BdSize	0.0188	0.0289
	(0.35)	(0.48)
Tqmed	−0.0397	−0.0992^*∗∗*^
	(−1.07)	(−2.30)
Cash	−0.00302	−0.0364^*∗∗∗*^
	(−0.85)	(−4.27)
Constant	0.351	0.334
	(1.33)	(1.16)
Observations	20749	20800
Year and industry	Yes	Yes
Pseudo *R*^2^	0.035	0.044
AIC	21517.6	36832.0
Log likelihood	−10666.8	−18316.0

^*∗*^ *p* < 0.10, ^*∗∗*^*p* < 0.05, and ^*∗∗∗*^*p* < 0.01.

## Data Availability

The dataset can be accessed upon request.
